# Validation of the EBMT Risk Score for South Brazilian Patients Submitted to Allogeneic Hematopoietic Stem Cell Transplantation

**DOI:** 10.1155/2013/565824

**Published:** 2013-12-12

**Authors:** Beatriz Stela Pitombeira, Alessandra Paz, Annelise Pezzi, Bruna Amorin, Vanessa Valim, Alvaro Laureano, Andrea Wieck, Lisandra Rigoni, Érica Ottoni, Gustavo Fisher, Liane Daudt, Lucia Silla

**Affiliations:** ^1^Hematology and Bone Marrow Transplantation, Clinical Hospital of Porto Alegre, 90035-903 Porto Alegre, RS, Brazil; ^2^Laboratory of Cell Culture and Molecular Analysis of Hematopoietic Cells, Clinical Hospital of Porto Alegre, 90035-903 Porto Alegre, RS, Brazil

## Abstract

*Background*. Allogeneic hematopoietic stem cell transplantation (HSCT) is still associated with a high transplant-related mortality rate. In 2009, the EBMT risk score was validated as a simple tool to predict the outcome after allogeneic HSCT for acquired hematological disorders. *Objectives*. The aim of this study was to validate the applicability of the EBMT risk score for allogeneic HSCT on South Brazilian patients. *Methods*. A retrospective observational study was performed based on patients' records and data base at Hospital de Clínicas de Porto Alegre, including all allogeneic transplants for malignant and severe aplastic anemia from 1994 to 2010. Patients were categorized according to EBMT risk score and overall survival (OS). Nonrelapse mortality (NRM) and relapse rate (RR) were analyzed. *Results*. There were 278 evaluable patients. OS, NRM, and RR at five years median followup were 48.7%, 40.7%, and 30.7%, respectively. The OS was 81.8% for risk score 0 and 0% for score 6 (*P* < 0.001), and NRM was 13.6% and 80% for risk scores 0 and 6, respectively (*P* = 0.001). *Conclusion*. The EBMT risk score can be utilized as a tool for clinical decision making before allogeneic HSCT for malignant hematological diseases and severe aplastic anemia at a single center in Brazil.

## 1. Introduction

Hematopoietic stem cell transplantation (HSCT) is a potentially curative therapy for a variety of malignant and nonmalignant hematological disorders [1, 2]. Since the first transplant, performed in 1939 [3], this procedure has evolved with advances in conditioning, HLA compatibility techniques, supportive care, management of complications, and expanding stem cells sources [2, 4–9]. As a result, indications for transplant and the pool of eligible patients have increased, notably allowing inclusion of elderly recipients [10] and increasing the annual number of unrelated HSCT, now exceeding 10.000 worldwide [11].

Allogeneic HSCT remains, however, associated with a significant risk of morbidity and mortality. This procedure can induce damage of various organs and tissues, from subclinical changes to life-threatening conditions [4, 12, 13], justifying the development of scores for the assessment of HSCT risk in a particular patient. On the other hand, nontransplant treatment strategies [14–17] have improved in recent years, particularly for chronic myeloid leukemia (CML).

In 1998, the EBMT (European Group for Blood and Marrow Transplantation) validated a simple risk score for patients submitted to allogeneic HSCT for CML based on five risk factors: age of recipient, disease stage, time interval from diagnosis to transplantation, donor type, and donor-recipient gender combination [18]. It is interesting to note that De Souza et al. [19] validated the EBMT score in a population of 1084 Brazilian CML patients. Later, in a retrospective analysis, the EBMT extended the score utility to a broad range of acquired hematological disorders, improving transplant risk assessment in general [17]. The latter was later tested and modified for different diseases and clinical situations [20–22].

The aim of this study was to validate the applicability of the EBMT risk score for allogeneic HSCT on South Brazilian patients with acquired hematological disorders from a single center at Hospital de Clínicas de Porto Alegre, Brazil.

## 2. Patients and Methods

### 2.1. Study Design and Database

A retrospective observational study was performed based on the review of patients' charts and database at the Hematology and Bone Marrow Transplantation Department of Hospital de Clínicas de Porto Alegre (HCPA), Brazil. This data holds information on 293 patients submitted to allogeneic HSCT performed between 1994 and 2010. Except for syngeneic donor transplants and HSCT performed for nonmalignant diseases other than aplastic anemia, all HLA-identical sibling and unrelated donor transplants were included.

### 2.2. Definitions of Risk Score and Endpoints

This analysis utilizes the original EBMT (European Group for Blood and Marrow Transplantation) risk score as reported and later adapted by Gratwohl et al. [17, 18], with five pretransplant risk factors: age of the patient, disease stage, time from diagnosis to transplant, donor type, and donor-recipient gender combination. The total risk score for an individual patient is the sum of points given to each variable, which can range from 0 to 7 points. Risk factors were distributed as age of recipient (0 for <20 years, 1 for 20–40 years, and 2 for >40 years); donor type (0 for HLA-identical sibling donor and 1 for unrelated donor); gender match (0 for all others and 1 for male recipient/female donor); time from diagnosis to transplantation (0 for <12 months and 1 for >12 months). Disease stage was categorized as early stage (0 point) including acute leukemia transplanted in first complete remission (CR), myelodysplastic syndrome (MDS) transplanted either untreated or in first CR, CML in first chronic phase, and lymphoproliferative disorders and multiple myeloma (MM) in first CR. Intermediate stage (1 point) includes acute leukemia in second CR, CML in all other stages except chronic phase and blast crisis, MDS in second CR or in partial remission, and lymphoproliferative disorders and MM in second CR, partial remission, or stable disease. Late stage disease (2 points) includes acute leukemia in all other disease stages, CML in blast crisis, MDS, lymphoproliferative disorders, and MM in all other stages than those previously defined. Disease stage in patients with severe aplastic anemia (SAA) was always considered as early stage (0 point). Donor and recipient CMV serologic status was also analyzed in our population.

Overall survival (OS), nonrelapse mortality (NRM), and relapse rate (RR) were the three endpoints analyzed in this study. Overall survival was defined as the probability of survival from transplant to death from any cause, with surviving patients censored at the last followup. NRM was defined as the probability of dying due to any cause other than disease recurrence and RR as the probability of disease recurrence [15].

### 2.3. Statistical Analysis

The calculations were done by SPSS v.18.0, and each endpoint was analyzed separately. Quantitative variables are described by median and range and categorical variables by frequencies and percentages. 95% confidence intervals were presented. Overall survival was estimated from the time of transplant using Kaplan-Meier curve method. NRM and relapse were calculated using cumulative incidence estimates. For each endpoint and each studied disease, a Cox regression model with a proportional hazards analysis was applied using the five risk factors. The predictive power of the EBMT risk score was calculated for total population and for each disease by computing the receiver operating characteristics area under the curve (ROC AUC) for each endpoint.

### 2.4. Ethical Aspects

This study was approved by the Ethics Committee of HCPA under number 09-607 and the data was analyzed anonymously according to Declaration of Helsinki for human studies.

## 3. Results

### 3.1. Patient Population

In total, 293 patients submitted to allogeneic HSCT were included. Of those, 16 patients were not eligible: eleven patients had nonmalignant disorders other than SAA and in 5 the information was not available.

From the 278 evaluable patients 156 were male (56%), with a median age of 32 years, ranging from 1 to 57 years. Fifty-nine patients had acute myeloid leukemia (AML), 48 ALL, 16 MDS, 76 CML, 27 lymphoproliferative disorders, 1 MM, and 51 SAA. Most procedures (238) were performed with HLA-identical sibling and 40 with HLA-matched and mismatched unrelated donors. Bone marrow was the stem-cell source in 220, peripheral blood in 52, and cord blood in 6 patients. Of the entire group of patients, 241 were submitted to myeloablative and 37 to reduced intensity conditioning regimens. Most transplants were performed at early disease stage (175 patients, 62.9%) and with more than 12 months from diagnosis. The sums of risk score points were 2 and 3 for the majority of patients (138 patients, 49.6%). No recipient had scored 7. The population characteristics are summarized in Table 1.

### 3.2. Overall Survival

**Figure 1:**
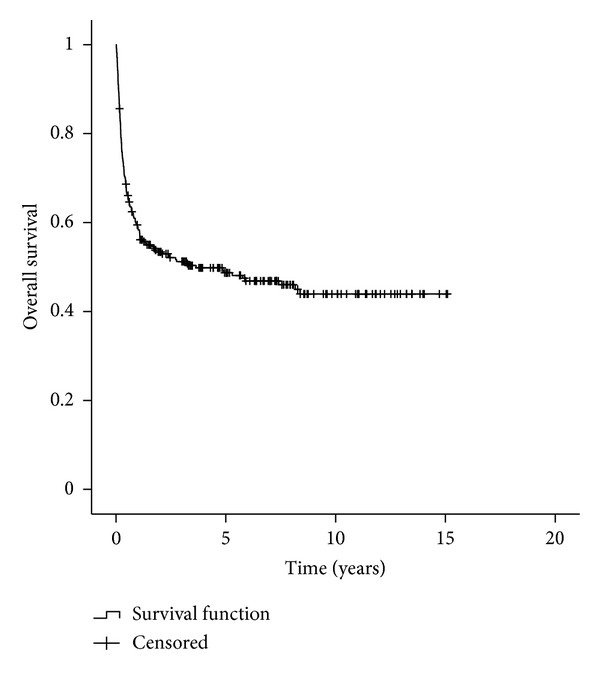
Overall survival.

The cumulative OS for the entire population was 48.7% at five years (95% confidence interval, 42.5%–54.9%) and 43.9% (95% CI, 37.1%–50.7%) at ten years. Median follow-up for surviving patients was 6.39 years, ranging from 58 days to 15 years (Figure 1).

**Figure 2:**
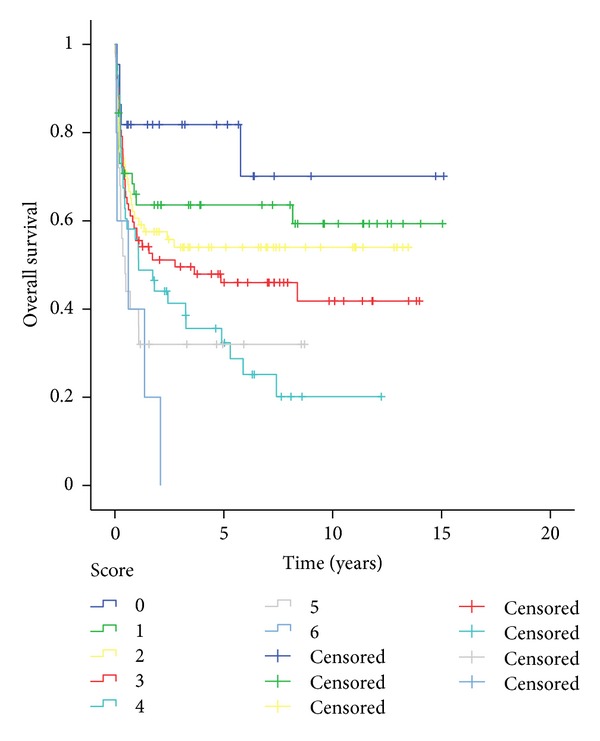
Overall survival on risk score.

There was a significant relation between the sum of risk score points and survival. Overall survival in five years decreases significantly from 81.8% on risk score 0 to 0% on score 6 (*P* < 0.001). Risk score 0 had a statistical difference when compared with scores 3, 4, 5, and 6 (*P* = 0.034; 0.003; 0.003; and <0.001, resp.). A significant difference was also observed between score 1 when compared to scores 4, 5, and 6, and scores 3 and 4 to score 6 (*P* = 0.010; 0.018; and 0.005, resp.) (Figure 2).

Some risk factors had a significant impact on survival for different diseases. For our total population, disease stage, time interval between disease diagnosis and HSCT, and donor type were the risk factors with significant impact on overall survival. Age, disease stage, and gender combination had significant impact only for AML, and donor type was statistically significant for AML and CML. The predictive power of the score for all diseases, analyzed by ROC AUC, was 0.667, ranging from 0.590 for SAA to 0.802 for AML. Proportional hazards were not performed to MDS, MM, and lymphoproliferative disorders because of the small number of patients. Hazard ratio on overall survival for all patients and for each disease is shown in Table 2.

### 3.3. Nonrelapse Mortality

**Figure 3:**
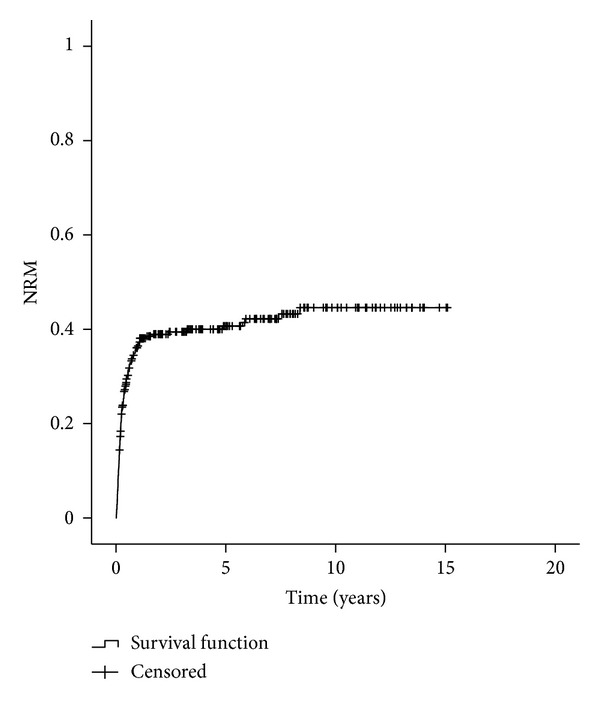
Nonrelapse mortality.

**Figure 4:**
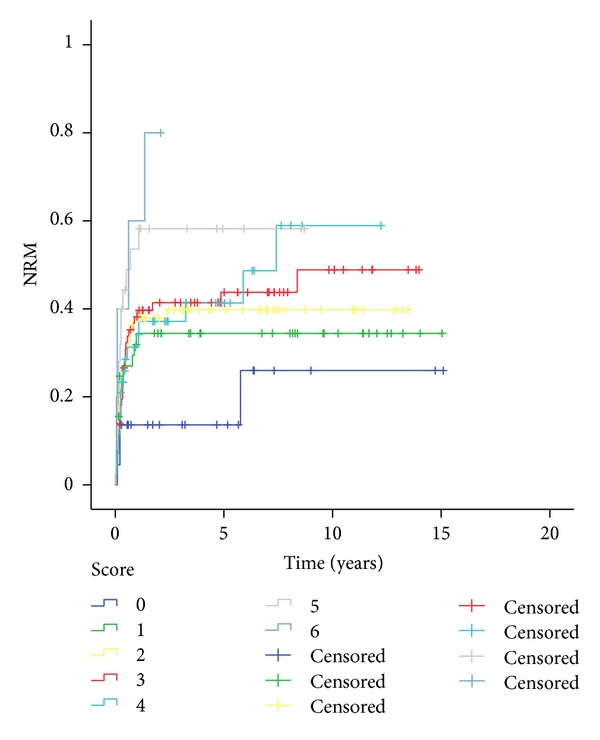
Nonrelapse mortality on risk score.

Cumulative nonrelapse mortality was 40.7% at five years (95% CI, 34.5%–46.9%) and 44.6% (95% CI, 37.6%–51.6%) at ten years (Figure 3). The NRM increased from 13.6% for risk score 0 to 80% for score 6 (*P* = 0.001). Risk score 0 was statistically different to scores 2, 3, 5, and 6 (*P* = 0.044; 0.034; 0.004; and 0.001, resp.), as score 1 to score 6 (*P* = 0.029) (Figure 4). Evaluating risk factors for NRM, advance disease stage (HR 3.8 (1.3–11.0)), and gender combination (HR 2.6 (1.1–6.4)) had significant difference for AML and donor type (HR 4.1 (1.7–10.2)) for CML. The predictive power for all diseases was 0.59, ranging from 0.55 for ALL to 0.77 for AML.

### 3.4. Relapse Rate

**Figure 5:**
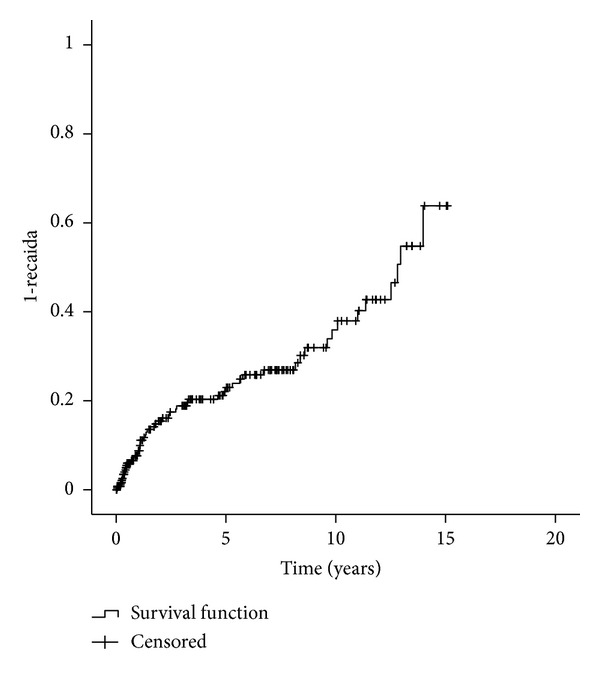
Deaths from relapse.

The RR of the entire population was 30.7% at five years (95% CI, 23.3%–38.1%) and 32.4% at ten years (95% CI, 24.4%–40.4%). For relapse, advanced disease stage had significant impact for ALL (HR 9.6 (2.6–36.0)), AML (HR 7.3 (2.2–24.0)), and CML (HR 5.1 (1.1–24.2)) and gender combination for ALL (HR 3.5 (1.2–10.1)). The predictive power for all diseases was 0.630, ranging from 0.47 for CML to 0.76 for ALL. Deaths from relapse were 22.1% at five years (95% CI, 15.5%–28.7%) and 28.5% at ten years (95% CI, 20.5%–36.5%) (Figure 5). The relation between relapse rate and prognostic scoring system was not statistically significant.

### 3.5. CMV Serologic Status

CMV serologic status was positive in 238 (86.5%) patients and there was no significant difference on overall survival between seropositive and seronegative recipients or between donor/recipient combinations of CMV serology (Table 1).

## 4. Discussion

Hematopoietic stem cell transplantation is a treatment modality able to cure many hematological disorders, but morbimortality rates remain high [18, 23, 24]. Efforts have been made trying to integrate pretransplant clinical variables into a single score in order to predict outcomes in HSCT recipients [25–27]. The aim of our study was to validate the 2009 EBMT risk score [17] in a single Brazilian center for all acquired hematological disorders.

Of the 278 patients, with a median age of 32 years, the majority (86.5%) had a positive serologic status for CMV, precluding statistical analysis, and were submitted to a matched-related, myeloablative allogeneic HSCT for an early stage disease. The median time interval from diagnosis to transplant was over 12 months, and the stem cell source more frequently used was bone marrow. OS, NRM, and death from relapse at five years were 48.7%, 40.7%, and 22.1%, respectively (Figures 1, 3, and 5). Although in a much smaller and different ethnic population of patients, precluding a strait forward comparison, our data differs from Gratwohl et al. [17] in time interval to transplant since the majority of the European patients were transplanted less than 12 months after diagnosis. The extended time to transplant observed in our population was probably due to a delay in referral to HSCT as previously shown in a Brazilian population [19].

Overall, the EBMT risk score has proved to be a reasonable tool to predict OS at our center. As shown by the EBMT group, patients with lower risk scores had a better OS after HSCT, and advanced disease stage, longer time interval from diagnosis to transplant, and unrelated donor were powerful predictors of worse survival in our population.

As for the stronger predictive value for AML (AUC: 0.802), observed in our group of patients, we could argue that, besides size sample, ours was a positively selected group of AML patients since the majority of patients were transplanted in first remission in a longer time interval to transplant. It is reasonable to speculate that for such long disease-free survival before transplant, patients were selected by attending a better remission with corresponding better prognosis [28]. As can be noted (Table 2), time interval to transplant was not significant for this as well as for any group of our patients. Recently, a modified EBMT risk score was proposed and validated to ALL and AML patients [20, 21], omitting time from diagnosis to transplantation since this parameter could be more susceptible to multiple sources of bias [1, 28], including a strong correlation with disease stage [21]. In addition, it is of note that most ALL patients in first CR were at high risk (Ph+ ALL and other cytogenetic unfavorable leukemias) (data not shown), whereas our CML patients were transplanted after over 12 months in first chronic phase which has been shown, as itself, to be predictive of a worse outcome [18]. These findings could explain why the predicted risk value was weaker, although in accordance to the EBMT findings [17], compared to our group of probably selected AML patients.

Finally, NRM and RR were also associated with risk scores categories but with a poorer predictive power than overall survival. Confirming the international literature [29], advanced disease stage was an independent risk factor associated with increased relapse rate in all malignancies (AML, ALL, and CML) evaluated in our study. Lymphoproliferative disorders, MDS, and MM had no sufficient number of patients to allow a reliable analysis. Although not considered as a risk factor at the EBMT score system, CMV serologic status, recognized as a negative risk factor for NRM and OS after HSCT [30], could not be evaluated in our sample of patients due to the very high incidence observed of CMV positivity.

In conclusion, here we demonstrated that the EBMT score can be utilized as a tool for clinical decision making for malignant and nonmalignant hematological disorders in a single center in Brazil. It should be noted that been retrospective and studying a consecutive cohort of patients from 1994 to 2010, its limitation is to have included mostly young and good risk patients. It will be necessary to test this predictive model prospectively since the present day population has a larger proportion of patients with more resistant diseases partially related to the use of newer agents or more extensive pretreatment with conventional drug combinations.

## Figures and Tables

**Table 1:** Patients distribution.

Category	AML	ALL	CML	MDS	MM	LD	SAA	Total
Sex								
Male	33 (55.9%)	30 (62.5%)	45 (59.2%)	6 (37.5%)	1 (100%)	12 (44.4%)	29 (56.9%)	156 (56.1%)
Female	26 (44.1%)	18 (37.5%)	31 (40.8%)	10 (62.5%)	0	15 (55.6%)	22 (43.1%)	122 (43.9%)

Source								
BM	49 (83.1%)	41 (85.4%)	63 (82.9%)	11 (68.8%)	0	7 (25.9%)	49 (96.1%)	220 (79.1%)
PBSC	10 (16.9%)	3 (6.3%)	13 (17.1%)	4 (25%)	1 (100%)	20 (74.1%)	1 (2%)	52 (18.7%)
CB	0	4 (8.3%)	0	1 (6.3%)	0	0	1 (2%)	6 (2.2%)

Regimen								
Myeloablative	58 (98.3%)	48 (100%)	76 (100%)	12 (75%)	0	0	46 (90.2%)	241 (86.7%)
RIC	1 (1.7%)	0	0	4 (25%)	1 (100%)	27 (100%)	5 (9.8%)	37 (13.3%)

Patient age								
Median	35	15	36.5	35	31	43	20	32
Range	1–55	1–47	7–57	2–55	31	18–56	5–51	1–57

Age								
<20	14 (23.7%)	27 (56.3%)	6 (7.9%)	4 (25%)	0	2 (7.4%)	26 (51%)	79 (28.4%)
20–40	27 (45.8%)	16 (33.3%)	40 (52.6%)	6 (37.5%)	1 (100%)	10 (37%)	19 (37.3%)	119 (42.8%)
>40	18 (30.5%)	5 (10.4%)	30 (39.5%)	6 (37.5%)	0	15 (55.6%)	6 (11.8%)	80 (28.8%)

Disease stage								
Early	28 (47.5%)	18 (37.5%)	60 (78.9%)	16 (100%)	0	2 (7.4%)	51 (100%)	175 (62.9%)
Intermediate	17 (28.8%)	23 (47.9%)	12 (15.8%)	0	1 (100%)	7 (25.9%)	—	60 (21.6%)
Advanced	14 (23.7%)	7 (14.6%)	4 (5.3%)	0	0	18 (66.7%)	—	43 (15.5%)

Time interval								
<12 months	24 (40.7%)	13 (27.1%)	13 (17.1%)	5 (31.3%)	0	1 (3.7%)	36 (70.6%)	92 (33.1%)
>12 months	35 (59.3%)	35 (72.9%)	63 (82.9%)	11 (68.7%)	1 (100%)	26 (96.3%)	15 (29.45)	186 (66.9%)

HLA								
HLA IS	50 (84.7%)	33 (68.8%)	69 (90.8%)	14 (87.5%)	1 (100%)	27 (100%)	44 (86.3%)	238 (85.6%)
Unrelated	9 (15.35)	15 (31.3%)	7 (9.2%)	2 (12.5%)	0	0	7 (13.7%)	40 (14.4%)

Gender com								
Other	42 (71.2%)	34 (70.8%)	58 (76.3%)	16 (100%)	0	21 (77.8%)	38 (74.5%)	209 (75.2%)
DF/RM	17 (28.8%)	14 (29.2%)	18 (23.7%)	0	1 (100%)	6 (22.2%)	13 (25.5%)	69 (24.8%)

Score								
0	2 (3.4%)	4 (8.3%)	1 (1.3%)	2 (12.5%)	0	0	13 (25.5%)	22 (7.9%)
1	10 (16.9%)	6 (12.5%)	7 (9.2%)	4 (25%)	0	0	18 (35.3%)	45 (16.2%)
2	17 (28.8%)	12 (25%)	20 (26.3%)	3 (18.8%)	0	1 (3.7%)	13 (25.5%)	66 (23.7%)
3	9 (15.3%)	12 (25%)	35 (46.1%)	7 (15.3%)	0	3 (11.1%)	6 (11.8%)	72 (25.9%)
4	10 (16.9%)	11 (22.9%)	9 (11.8%)	0	1 (100%)	11 (40.7%)	1 (2.0%)	43 (15.5%)
5	8 (13.6%)	2 (4.2%)	4 (5.3%)	0	0	11 (40.7%)	0	25 (9.0%)
6	3 (5.1%)	1 (2.1%)	0	0	0	1 (3.7%)	0	5 (1.8%)

CMV								
Recipient pos	52 (91.2%)	37 (77.1%)	65 (85.5%)	14 (87.5%)	1 (100%)	23 (88.5%)	46 (90.2%)	238 (86.5%)
Recipient neg	5 (8.8%)	11 (22.9%)	11 (14.5%)	2 (12.5%)	0	3 (11.5%)	5 (9.8%)	37 (13.5%)

AML: acute myeloid leukemia; ALL: acute lymphoblastic leukemia; CML: chronic myeloid leukemia; MDS: myelodysplastic syndrome; MM: multiple myeloma; LD: lymphoproliferative disorders; SAA: severe aplastic anemia; BM: bone marrow; PBSC: peripheral blood stem cell; CB: cord blood; HLA IS: human leukocyte antigen identical sibling; Gender com: gender combination between donor and recipient; DF/RM: donor female/recipient male; CMV: cytomegalovirus; neg: negative; pos: positive.

**Table 2:** Impact of pretransplant risk factors on overall survival.

Risk factors	AML	ALL	CML	AA	Total
Age class					
<20	1	1	1	1	1
20–40	3.4 (1.0–11.7)	1.3 (0.6–2.8)	0.9 (0.3–3.0)	1.7 (0.7–4.5)	1.4 (0.9–2.2)
>40	3.8 (1.1–13.7)	1.4 (0.5–3.9)	1.1 (0.3–3.9)	1.1 (0.2–5.3)	1.4 (0.9–2.2)

Disease stage					
Early	1	1	1	1	1
Intermediate	2.1 (0.8–5.4)	1.4 (0.6–3.0)	1.9 (0.9–4.0)	—	1.5 (1.0–2.2)
Advanced	5.1 (2.1–12.8)	2.4 (0.9–6.3)	2.1 (0.6–6.8)	—	2.0 (1.3–3.1)

Time interval					
<12 months	1	1	1	1	1
>12 months	1.8 (0.8–3.8)	0.8 (0.4–1.8)	1.7 (0.7–4.3)	1.4 (0.5–3.5)	1.5 (1.1–2.2)

Donor type					
HLA	1	1	1	1	1
Other	3.0 (1.3–7.1)	1.6 (0.8–3.3)	3.6 (1.5–8.6)	1.7 (0.6–5.2)	2.4 (1.6–3.6)

Gender combination					
Other	1	1	1	1	1
DF/RM	2.4 (1.2–5.1)	1.9 (0.9–4.0)	0.7 (0.3–1.4)	0.8 (0.3–2.3)	1.1 (0.8–1.7)

ROC curve					
AUC	0.802	0.743	0.631	0.590	0.667

AML: acute myeloid leukemia; ALL: acute lymphoblastic leukemia; CML: chronic myeloid leukemia; HLA IS: human leukocyte antigen identical sibling; DF/RM: donor female/recipient male; ROC AUC: receiver operating characteristics area under the curve.

## References

[1] Copelan EA (2006). Hematopoietic stem-cell transplantation. *New England Journal of Medicine*.

[2] Gyurkocza B, Rezvani A, Storb RF (2010). Allogeneic hematopoietic cell transplantation: the state of the art. *Expert Review of Hematology*.

[3] Lorenz E, Uphoff D, Reid TR (1951). Modification of irradiation injury in mice and guinea pigs by bone marrow injections. *Journal of the National Cancer Institute*.

[4] Solh M, Arat M, Cao Q, Majhail NS, Weisdorf D (2011). Late-onset noninfectious pulmonary complications in adult allogeneic hematopoietic cell transplant recipients. *Transplantation*.

[5] Hamadani M, Craig M, Awan FT, Devine SM (2010). How we approach patient evaluation for hematopoietic stem cell transplantation. *Bone Marrow Transplantation*.

[6] Hurley CK (2002). HLA diversity: detection and impact on unrelated hematopoietic stem cell donor characterization and selection. *International Journal of Hematology*.

[7] Shaw BE, Veys P, Pagliuca A (2009). Recommendations for a standard UK approach to incorporating umbilical cord blood into clinical transplantation practice: conditioning protocols and donor selection algorithms. *Bone Marrow Transplantation*.

[8] Bray RA, Hurley CK, Kamani NR (2008). National marrow donor program HLA matching guidelines for unrelated adult donor hematopoietic cell transplants. *Biology of Blood and Marrow Transplantation*.

[9] Aversa F, Velardi A, Tabilio A, Reisner Y, Martelli MF (2001). Haploidentical stem cell transplantation in leukemia. *Blood Reviews*.

[10] Bregni M, Herr W, Blaise D (2011). Allogeneic stem cell transplantation for renal cell carcinoma. *Expert Review of Anticancer Therapy*.

[11] King RJ, Confer DL, Greinix HT (2011). Unrelated hematopoietic stem cell donors as research subjects. *Bone Marrow Transplantation*.

[12] Roziakova L, Mladosievicova B (2010). Endocrine late effects after hematopoietic stem cell transplantation. *Oncology Research*.

[13] Pidala J, Kim J, Anasetti C (2011). The global severity of chronic graft-versus-host disease, determined by national institutes of health consensus criteria, is associated with overall survival and non-relapse mortality. *Haematologica*.

[14] Burnett AK, Knapper S (2008). *Chapter 3. In: Hematopoietic Stem Cell Transplantation in Clinical Practice, 1e*.

[15] Varaldo R, Frassoni F (2008). *The EBMT Handbook: Hematopoietic Stem Cell Transplantation*.

[16] Döhner H, Estey EH, Amadori S (2010). Diagnosis and management of acute myeloid leukemia in adults: recommendations from an international expert panel, on behalf of the European LeukemiaNet. *Blood*.

[17] Gratwohl A, Stern M, Brand R (2009). Risk score for outcome after allogeneic hematopoietic stem cell transplantation: a retrospective analysis. *Cancer*.

[18] Gratwohl A, Hermans J, Goldman JM (1998). Risk assessment for patients with chronic myeloid leukaemia before allogeneic blood or marrow transplantation. *The Lancet*.

[19] De Souza CA, Vigorito AC, Ruiz MA (2005). Validation of the EBMT risk score in chronic myeloid leukemia in Brazil and allogeneic transplant outcome. *Haematologica*.

[20] Hemmati PG, Terwey TH, Le Coutre P (2011). A modified EBMT risk score predicts the outcome of patients with acute myeloid leukemia receiving allogeneic stem cell transplants. *European Journal of Haematology*.

[21] Terwey TH, Hemmati PG, Martus P (2010). A modified EBMT risk score and the hematopoietic cell transplantation-specific comorbidity index for pre-transplant risk assessment in adult acute lymphoblastic leukemia. *Haematologica*.

[22] Rezvani K, Kanfer EJ, Marin D (2012). EBMT risk score predicts outcome of allogeneic hematopoietic stem cell transplantation in patients who have failed a previous transplantation procedure. *Biology of Blood and Marrow Transplantation*.

[23] Pasquini MC, Wang Z, Horowitz MM, Gale RP (2010). 2010 report from the Center for International Blood and Marrow Transplant Research (CIBMTR): current uses and outcomes of hematopoietic cell transplants for blood and bone marrow disorders. *Clinical Transplants*.

[24] Cooper LJN (2009). New approaches to allogeneic hematopoietic stem cell transplantation in pediatric cancers. *Current Oncology Reports*.

[25] Parimon T, Au DH, Martin PJ, Chien JW (2006). A risk score for mortality after allogeneic hematopoietic cell transplantation. *Annals of Internal Medicine*.

[26] Sorror ML, Maris MB, Storb R (2005). Hematopoietic cell transplantation (HCT)-specific comorbidity index: a new tool for risk assessment before allogeneic HCT. *Blood*.

[27] Xhaard A, Porcher R, Chien JW (2008). Impact of comorbidity indexes on non-relapse mortality. *Leukemia*.

[28] Gratwohl A (2012). The EBMT risk score. *Bone Marrow Transplantation*.

[29] Allogeneic bone marrow transplantation for leukaemia in Europe (1988). Report from the working party on leukaemia, European Group for Bone Marrow Transplantation. *The Lancet*.

[30] Boeckh M, Ljungman P (2009). How I treat cytomegalovirus in hematopoietic cell transplant recipients. *Blood*.

